# The 2024 ISCB Innovator Award—Dr Su-In Lee

**DOI:** 10.1093/bioinformatics/btae287

**Published:** 2024-06-28

**Authors:** Mallory L Wiper

**Affiliations:** The International Society for Computational Biology


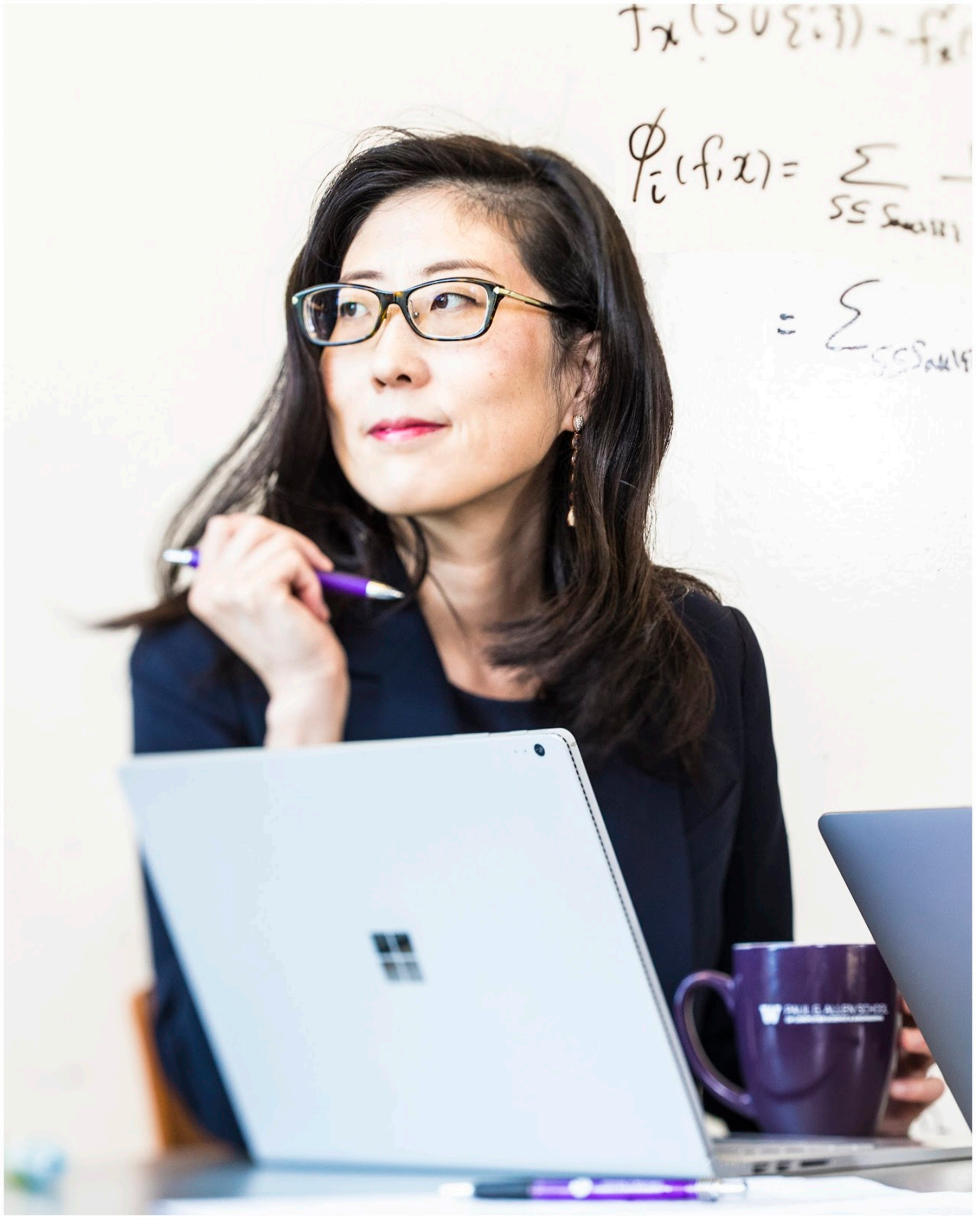


The ISCB Innovator Award is presented annually to a leading scientist who not only makes progressive contributions to computational biology, but who also consistently pursues unexplored directions in the field. This year, at the 32nd Annual Intelligent Systems for Molecular Biology conference (ISMB), the International Society for Computational Biology has the honor of presenting the Innovator Award to Dr Su-In Lee, the recent winner of the 2024 Samsung Ho-Am Prize, the Korean Nobel Prize.

## Early inspirations and the shift to computational biology

Dr Lee’s affinity for math and science began from a young age, in large part thanks to her father and his background in shipbuilding engineering. His profession fascinated her because of the output of his work—designing large ships capable of staying afloat and moving quickly through water—an output requiring a deep understanding of several areas, including physics and fluid mechanics. But her father’s influence on her interests didn’t stop there! Not only did he play a key role in fostering her love of science, but he was instrumental in her love of mathematics, too, having taught her math, like calculus and geometry. These lessons, in turn, nurtured her love of mathematical patterns and theorems.

From this love of patterns and theorems, Lee’s interest in math grew, leading her to immerse herself in the world of AI research during her undergraduate education in Korea. Her curiosity in this relatively new field led her to conduct her undergraduate thesis**—**for which she won the Best Undergraduate Thesis Award from Samsung**—**on developing a deep neural network (DNN) that could recognize handwritten digits. From this project, Lee was inspired to use DNNs and other similar models to understand the bigger questions surrounding human cognition and how the brain works.

When Dr Lee started her PhD at the Stanford AI Lab, however, her research pursuits changed course once more. The emerging microarray technology being used in molecular biology and genetics, and the accompanying computational challenges presented therein, captivated her, effectively marking the beginning of her exploration into computational biology.

When she accepted a faculty position at the University of Washington, the scope of Lee’s research broadened to include AI applications for clinical medicine, where AI interpretability and transparency were focal points. Presently, her lab operates at an intersecting point of the ABC fields upon which the future of medicine hinges: AI, biology, and clinical medicine.

## Influential mentors

Looking back on her academic career, Lee notes that she has been surrounded by *many* inspiring and influential people. There have been so many influential people, in fact, that narrowing down a singular most influential figure is a difficult task. But Lee does have a few people that come to mind who have significantly impacted her academic career toward and within computational biology.

One very influential individual for Lee was her undergraduate thesis advisor Dr Soo-Young Lee at the Korea Advanced Institute of Science and Technology. Dr Soo-Young Lee introduced her to the world of AI and subsequently to the intricate world of deep neural networks. This early introduction to AI laid much of the groundwork for Lee’s future endeavors!

When Lee continued into her PhD, her advisor Dr Daphne Koller, a prominent AI researcher, was responsible for introducing her to the field of computational biology. In fact, Lee was one of Koller’s early students to study AI *and* biology, specifically exploring AI’s usage and usefulness in addressing biological questions.

Following her PhD years, Lee cites Dr Aviv Regev, a former professor at MIT and the Broad Institute, as having been an encouraging and inspirational figure since Lee’s early faculty career as a computational biologist and setting an extraordinary example of leadership and the importance of interdisciplinary collaboration.

## Research and evolving pursuits

When it comes to her research, Lee has stated that one of the most unexpected findings so far was the “critical importance of model interpretability and explainability.” Specifically, it was the *depth* of the impact of interpretability and explainability that became increasingly evident as work in Lee’s lab progressed. Because of this revelation, Lee’s research group took a unique approach to developing clinical AI models by prioritizing interpretability, leading to the development of SHAP, an influential and widely used explainable AI framework.

Delving into AI interpretability ultimately reshaped Lee’s research pursuits. What was previously a side topic of interest became an exciting focal area of research!

With respect to current areas of interest, Lee has said that her research into AI auditing frameworks stands out most. This work utilizes explainable AI to audit existing AI models to uncover processing flaws. A large part of this research is due to the discovery of concerning trends in COVID AI prediction and classification models. Specifically, AI’s predictions were getting things wrong because it was relying on shortcuts. Lee says she’s drawn to the area of AI model auditing because it underscores the importance of understanding the reasoning processes of different AI models so that such models aren’t blindly used and implicitly trusted. Having a more thorough understanding of the reasoning processes is something that, while useful in clinical medicine, can also be useful in many other disciplines including computational biology.

## Training and mentoring new scientists

From the point of view of a Principal Investigator (PI), Lee has gained a deeper understand of research as a whole through the more broad perspective of research that a PI has to take. For instance, a PI must oversee project direction, manage resources, recruit collaborators, find funding, promote the lab’s research, and guide trainees—the latter point being the most important part of the job, according to Lee!

Before coming to computational biology, Lee initially pursued computer science and electrical engineering. The transition to biology and medicine solidified for her the importance of being willing to tread into unfamiliar territory and embrace new challenges by learning unfamiliar concepts and tackling problems in a different field. These lessons are ones that Lee tries to instill in her own students. She encourages them to explore different areas of research and to take a fearless approach to learning new things! When students express doubt in themselves or their knowledge in biology, she reminds them that they *are* biologists and that a fearless approach to learning, both within your own field of study and in new ones, can lead to some of most meaningful scientific contributions.

## Reflections on the ISCB innovator award

On being the recipient of the 2024 ISCB Innovator Award, Lee said, “it’s an incredible honor for me,” and that she’s very grateful for this recognition. She noted, too, that there are so many outstanding researchers in the field that it seems a nearly impossible task to select just one, but she is truly humbled to have been chosen as this year’s winner.

