# Correction to: The 2024 ISCB Innovator Award—Dr Su-In Lee

**DOI:** 10.1093/bioinformatics/btae460

**Published:** 2024-07-31

This is a correction to: Mallory L Wiper, The 2024 ISCB Innovator Award—Dr Su-In Lee, *Bioinformatics*, Volume 40, Issue Supplement_1, July 2024, Pages i5–i6, https://doi.org/10.1093/bioinformatics/btae287

In the originally published version of this paper Dr Su-In Lee’s name was given incorrectly in the title. This error has been corrected.

